# Sequence variation of necdin gene in Bovidae

**DOI:** 10.1186/s40781-018-0191-7

**Published:** 2018-12-20

**Authors:** Sunday O. Peters, Marcos De Donato, Tanveer Hussain, Hectorina Rodulfo, Masroor E. Babar, Ikhide G. Imumorin

**Affiliations:** 1 Department of Animal Science, Berry College, Mount Berry, GA 30149 USA; 2 Tecnologico de Monterrey, Escuela de Ingenieria y Ciencias, Ave. Epigmenio Gonzalez 500, 76130 Queretaro, CP Mexico; 3 Department of Molecular Biology, Virtual University of Pakistan, Lahore, 54000 Pakistan; 4 African Institute for Biosciences Research and Training, Ibadan, Nigeria; 5 School of Biological Sciences, Georgia Institute of Technology, Atlanta, 30332 USA

**Keywords:** MAGE, Bovini, Caprini, Phylogenetics

## Abstract

**Background:**

Necdin (NDN), a member of the melanoma antigen family showing imprinted pattern of expression, has been implicated as causing Prader-Willi symptoms, and known to participate in cellular growth, cellular migration and differentiation. The region where NDN is located has been associated to QTLs affecting reproduction and early growth in cattle, but location and functional analysis of the molecular mechanisms have not been established.

**Methods:**

Here we report the sequence variation of the entire coding sequence from 72 samples of cattle, yak, buffalo, goat and sheep, and discuss its variation in Bovidae. Median-joining network analysis was used to analyze the variation found in the species. Synonymous and non-synonymous substitution rates were determined for the analysis of all the polymorphic sites. Phylogenetic analysis were carried out among the species of Bovidae to reconstruct their relationships.

**Results:**

From the phylogenetic analysis with the consensus sequences of the studied Bovidae species, we found that only 11 of the 26 nucleotide changes that differentiate them produced amino acid changes. All the SNPs found in the cattle breeds were novel and showed similar percentages of nucleotides with non-synonymous substitutions at the N-terminal, MHD and C-terminal (12.3, 12.8 and 12.5%, respectively), and were much higher than the percentage of synonymous substitutions (2.5, 2.6 and 4.9%, respectively). Three mutations in cattle and one in sheep, detected in heterozygous individuals were predicted to be deleterious. Additionally, the analysis of the biochemical characteristics in the most common form of the proteins in each species show very little difference in molecular weight, pI, net charge, instability index, aliphatic index and GRAVY (Table 4) in the Bovidae species, except for sheep, which had a higher molecular weight, instability index and GRAVY.

**Conclusions:**

There is sufficient variation in this gene within and among the studied species, and because NDN carry key functions in the organism, it can have effects in economically important traits in the production of these species. NDN sequence is phylogenetically informative in this group, thus we propose this gene as a phylogenetic marker to study the evolution and conservation in Bovidae.

## Background

Cetartiodactyla is one of the most diversified mammalian orders, with 330 living species group into 128 genera [1]. This group contains whales, dolphins, hippopotamus, antelopes, deer, cattle, buffalo, sheep, goat, giraffes, camels, pigs, among many others [2]. Both morphological and molecular studies have supported the monophyletic evolution of five groups within Cetartiodactyla: (1) Cetacea, (2), Hippopotamidae, (3) Tylopoda, (4) Suina (containing Suidae and Tayassuidae) and (5) Ruminantia, which includes the infraorders Tragulina and Pecora, which in turn includes the 5 families: Giraffidae, Cervidae, Moschidae, Antilocaprinae and Bovidae [3–6]. Of these, members of the Bovidae family are the most diversified, with 142 species, including cattle, buffalo, sheep and goat [7].

Necdin (NDN), a member of the melanoma antigen (MAGE) family which is comprised of over 60 genes that share the highly-conserved MAGE homology domain (MHD) [8], is one of five genes located in the deletion region of the Prader-Willi syndrome in human, a rare genetic neurodevelopmental disease characterized by a variety of physical, cognitive, and behavioral defects [9], including short stature, early-childhood onset obesity, developmental delay/mild intellectual disability; growth hormone insufficiency, low muscle tone, increased food intake, low levels of insulin and insulin-like growth factor 1 (IGF1), incomplete sexual development, hypogonadism, and male infertility [10]. Gene inactivation studies in mouse suggest that NDN is responsible for the specific Prader-Willi symptoms [11]. It is the best characterized MAGE gene implicated as a negative growth regulator, and proposed to participate in a broad range of biological activities including cell growth, migration, differentiation and cell death/survival, but the precise molecular function is largely unknown [12].

NDN, as well as MAGEL2, shows preferential paternal expression (imprinting) being highly expressed in whole brain, hypothalamus, amygdala and pituitary gland, moderately expressed in adipocytes, uterus, lung, adrenal gland, placenta and smooth muscle [13]. NDN has been implicated as a transcription factor which acts activating the gonadotropin releasing hormone (GNRH1) in immature migratory cells, binding to the MSX1 transcription factor, which is a repressor of GNRH1 [14]. It has been shown as an imprinted tumor suppressor gene which affects cancer cell motility, invasion and growth in ovarian, prostate, urothelial, neck and head cancers [15]. Furthermore, NDN has shown to interact with E2F4 and E2F1 in the control of gene transcription [16] as well as to inhibit PPARγ1 gene expression providing a potential molecular mechanism through which it regulates adipogenesis [17].

The imprinting patter of expression, a type of monoallelic pattern of expression, has been shown to increases evolvability, by facilitating positive Darwinian selection on heterozygous individuals, and by simultaneously allowing a relaxation of purifying selection (also known as negative selection) on heterozygous individuals [18]. In addition, monoallelic expression has shown to be an important evolutionary mechanism for the maintenance of genetic diversity and gene diversification [19]. Thus, NDN could be potentially informative for phylogenetic analysis to study groups that have shown to be difficult to resolve.

In addition, in the proximal region of bovine chromosome 21, where NDN is located, several QTLs have been associated, including calving ease (the percentage of unassisted births in first-calf heifers) [20–22], gestation length [21, 23], scrotal circumference (used as a major selection criterion to improve precocity and fertility) [24] and birth weight [22], among others.

In this study, we sequenced the coding region of the NDN gene in members of the Bovidae family, as potentially associated to traits with economic impact in the domestic species used for food production, clothing materials (hear, wool, leather) and to carry out heavy labor, due to its role in growth and neuronal development, and SNPs found here can be used for association studies to economically important traits in cattle, yak, sheep, goat and buffalo. This gene can also be used as a marker to study the evolution and conservation of this family, which have shown a rapid diversification.

## Methods

The program LAGAN of the mVISTA suite (http://genome.lbl.gov/vista/index.shtml) [25] was used for the multiple comparison of the published genomic sequences in cattle (AC_000178:735683–738,292, Taxon ID: 9913), sheep (NW_011943091:477276–478,942, Taxon ID: 9938), yak (NW_005392936:256281–257,942, Taxon ID: 72004), goat (NC_030828:463679–465,342, Taxon ID: 9925), water buffalo (NW_005785325:305827–307,494, Taxon ID: 89462), bison (NW_011494393, Taxon ID: 43346), as well as pig (NC_010443.4:212656–216,850, Taxon ID: 9823) and Arabian camel (NW_011591329:665988–667,365, Taxon ID: 9838) as outgroups. To determine the identity of the sequences, a mobile window of 100 nucleotides was used. The resulting alignment was used to select the most conserved sequences within the gene and 500 base pairs upstream and downstream for designing a set of primers that can amplify in multiple species. The online program Primer3Plus (version 2, http://www.bioinformatics.nl/cgi-bin/primer3plus/primer3plus.cgi/) [26] was used to designed the primers with the reference sequence for cattle. The designed primers were tested with genomic DNA from cattle, sheep, goat, yak, buffalo and pig, to determine which set would produce bright bands without unspecific products.

Genomic DNA samples from the collection of the Animal Breeding, Genetics and Genomics Laboratory at the International Programs, College of Agriculture and Life Sciences, Cornell University, were used to amplify a DNA fragment of 1283 bp in 49 samples of 18 different breeds of cattle, as well as 3 individual yak, 4 samples of 3 breeds of river buffalo, 10 of 4 breeds of sheep and 6 samples of 4 breeds of goat (Table 1). For PCR, we amplify a final volume of 20 μL, with 1.5 mM MgCl2, 100 μM of each dNTP, 0.2 μM of each oligonucleotide and 1 U of Taq DNA polymerase (Syd Labs Inc., Malden, MA). The amplification was carried out as follows: an initial denaturation at 94 °C (4 min), followed by 35 cycles of denaturation at 94 °C (30 s), annealing at 55 °C (45 s), and extensions at 72 °C (1 min), finalizing with an extension at 72 °C (10 min).

**Table 1:** Species and breeds of Bovidae with the sequence of NDN gene studied and the accession numbers of all the sequences published in the GenBank

Breed	Species	Number	Country	Accession Numbers
Achai	Indicine cattle	3	Pakistan	JX196877-JX196879
Angus	Taurine cattle	3	USA	JX196880-JX196882
Bhagnari	Indicine cattle	3	Pakistan	JX196883-JX196885
Brangus	Indicine x taurine cattle	3	USA	JX196886-JX196888
Cholistani	Indicine cattle	3	Pakistan	JX196889-JX196891
Dajal	Indicine cattle	2	Pakistan	JX196892-JX196893
Dhanni	Indicine cattle	3	Pakistan	JX196894-JX196896
Hereford	Taurine cattle	3	USA	JX196897-JX196899
Holstein	Taurine cattle	3	USA	JX196900-JX196902
Lohani	Indicine cattle	2	Pakistan	JX196903-JX196904
Nari Master	Indicine x taurine cattle	3	Pakistan	JX196905-JX196907
N’Dama	African cattle	2	Nigeria	JX196908-JX196909
Muturu	African cattle	2	Nigeria	JX196910-JX196911
Red Sindhi	Indicine cattle	3	Pakistan	JX196912-JX196914
Sahiwal	Indicine cattle	3	Pakistan	JX196915-JX196917
Sokoto Gudali	Indicine cattle	2	Nigeria	JX196918-JX196919
Tharparker	Indicine cattle	3	Pakistan	JX196920-JX196922
White Fulani	Indicine cattle	3	Nigeria	JX196923-JX196925
Yak	B. gruniens	3	Pakistan	JX196926-JX196928
Nili-Ravi	*B. bubalis*	1	Pakistan	JX196873
Ravi	B. bubalis	2	Pakistan	JX196874 JX196875
Nili	B. bubalis	1	Pakistan	JX196876
Kajli	*O. aries*	1	Pakistan	JX196929
Finn	O. aries	6	USA	JX196930-JX196932 JX196935-JX196937
Dorset	O. aries	2	USA	JX196933 JX196934
WAD	O. aries	1	Nigeria	JX196938
Beetal	*C. hircus*	1	Pakistan	JX196939
White	C. hircus	1	Pakistan	JX196940
WAD	C. hircus	2	Nigeria	JX196941 JX196942
Red Sokoto	C. hircus	2	Nigeria	JX196943 JX196944

PCR products were detected on 2.0% agarose gel including a dilution of 1:10,000 of GelRed Nucleic Acid Stain (Biotium, CA, USA) and compared to GENEMate Quanti-Marker 100 bp DNA ladder (BioExpress, UT, USA) for size estimation. Cycle-sequencing of the amplified fragments was carried out on the Applied Biosystems Automated 3730XL DNA Analyzer using Big Dye Terminator (Applied Biosystems, CA, USA) chemistry and AmpliTaq-FS DNA Polymerase. The sequence was visualized using CodonCode Aligner (V 3.5, CodonCode Corporation, MA, USA) to assess the quality and identify their differences. We did a BLAST search to find homologous sequences in other mammalian species. We use genomic sequences from cattle (AC_000178:736183–737,792) as reference to compared to the NDN sequences obtained in this study from the Bovidae species.

Median-joining network algorithm, which allows multi-state data, was used for the genomic sequences of the NDN genes by the Network software (version 4.6.1.0, www.fluxus-engineering.com). Synonymous and non-synonymous substitution rates corrected for multiple substitutions were determined using the SNAP program [27]. The deleterious effect of the non-synonymous SNPs found were estimated using the web server PROVEAN (Protein Variation Effect Analyzer, http://provean.jcvi.org), which is a software tool that uses a sequence-based prediction algorithm to determine whether an amino acid substitution has an impact on the biological function of a protein [28]. This algorithm allows for the best-balanced separation between the deleterious and neutral amino acids, based on a threshold. The score < − 2.5 indicates that the variant is deleterious and > − 2.5 score is considered as a neutral variant. ProtParam (https://web.expasy.org/protparam), which is a tool use for the computation of the molecular weight, theoretical pI, protein net charge, instability index, aliphatic index and grand average of hydropathicity (GRAVY) [29].

The evolutionary history of the Bovidae species was inferred using the Maximum Likelihood method based on the Tamura-Nei model [30], conducted in MEGA7 [31]. A bootstrap test [32] of 1000 replicates was used to determine the statistical support of the branches in the most likely tree. Initial tree(s) for the heuristic search were obtained automatically by applying Neighbor-Join and BioNJ algorithms to a matrix of pairwise distances estimated using the Maximum Composite Likelihood (MCL) approach, and then selecting the topology with superior log likelihood value. A discrete Gamma distribution was used to model evolutionary rate differences among sites (5 categories (+G, parameter = 0.5094)).

In addition, a Bayesian phylogenetic analysis was conducted using Mr.Bayes, v 3.2.1 [33], implementing the general time-reversible (GTR) model with the rate at each site as random variable with a gamma distribution (G) and a proportion of invariable sites. Markov chain Monte Carlo (MCMC) chains were carried out for 10,000,000 generations.

## Results

**Fig. 1:**
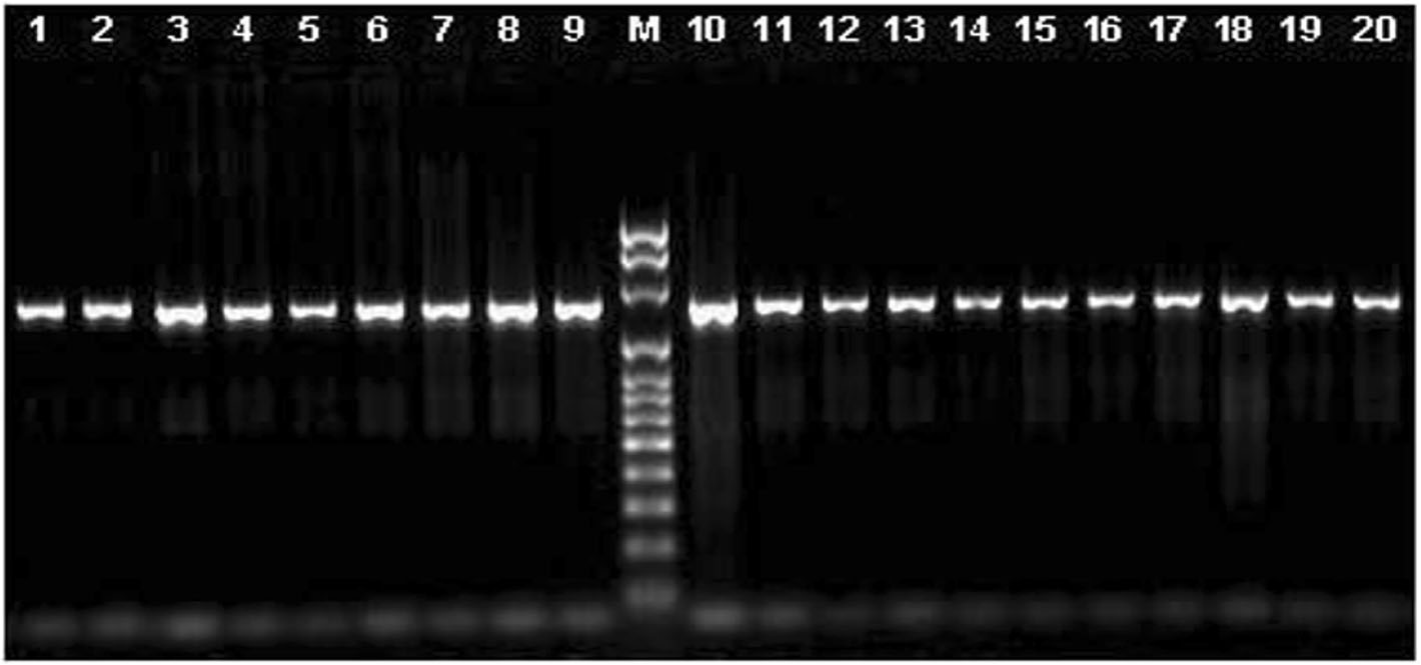
PCR amplification of the 1283 bp fragment containing the coding region of the Necdin (NDN) gene in cattle (lines 1–9) yak (lines 10–11) buffalo (lines 12–14) goats (lines 15–17) and sheep (lines 18–20) run in a 1.5% agarose gel and stained with GelRed (Phenix Research Products Candler NC). M: Molecular weight marker 100pb (GENEMate Quanti-Marker UT)

**Fig. 2:**
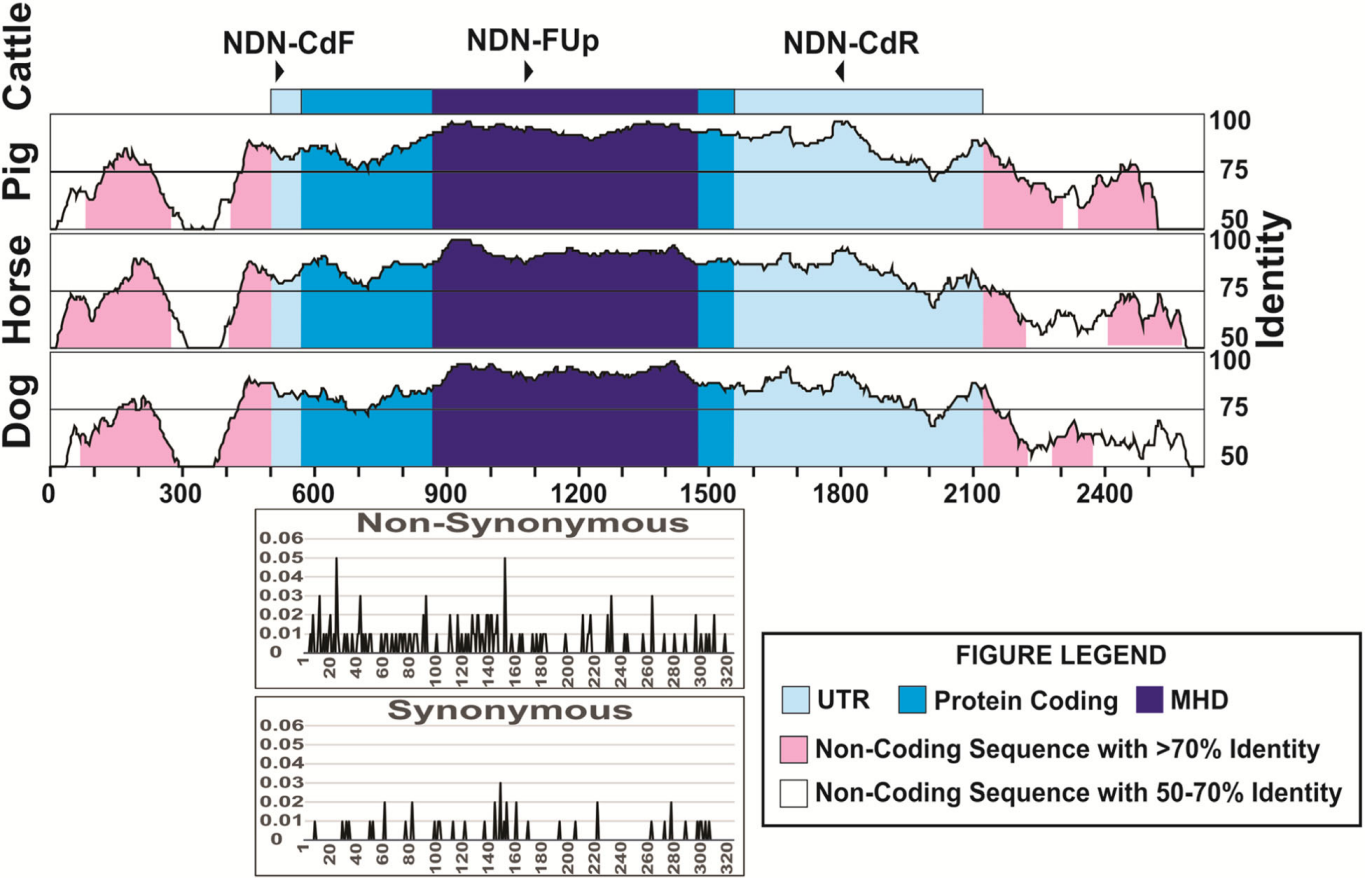
Structure of the Necdin gene showing the sequence conservation for pig, horse and dog using the program suite mVISTA. The primers used in this study are shown as arrowheads oriented from 5′ to 3′ and the MAGE homology domain (MHD) is also shown. Graphs of the synonymous and non-synonymous substitution rates, from the SNPs published on GeneBank and the variants found here, are shown

We used several sets of primers but only the pair FCd/RCd (Table 2) produced a single fragment of 1283 bp in all the species tested, except for pig, (Fig. 1). The amplified fragment contained the entire coding region, and those primers were located in the most conserved regions in the 5′ and 3’ UTRs (Fig. 2). The primers designed on the promoter regions produced unspecific fragments, even though this region is highly conserved. The primers selected here for the amplification of the coding region and its sequencing (Table 2) seem to be useful to study this gene sequence in all species from the five families of Ruminantia. In fact, primers specific for the other groups of Cetartiodactyla can be obtained to study the gene sequence in other species. Using the three primers we obtained good quality sequence for 1194 bp in all the samples analyzed, which included the entire coding region, plus 15 bp upstream and 200 bp downstream.

**Table 2:** Nucleotide changes in the sequence of the oligonucleotides used as primers for PCR and sequencing compared to the sequences in several species of Cetartiodactyla analyzed in this study

Gene	NDN-FCd	NDN-FUp	NDN-RCd
Cattle	GGAAAGCAGACTCGAAGAGC	GAGTTTTCGCTGGTCAAAGC	GCTTTCGCTTTTGTGCTACC
Yak	GGAAAGCAGACTCGAAGAGC	GAGTTTTCGCTGGTCAAAGC	GCTTTCGCTTTTGTGCTACC
Bison	GGAAAGCAGACTCGAAGAGC	GAGTTTTCGCTGGTCAAAGC	GCTTTCGCTTTTGTGCTACC
Buffalo	GGAAAGCAGACTCGAAGAGC	GAGTTTTCGCTGGTCAAAGC	GCTTTCGCTTTTGTGCTACC
Goat	GGAAAGCAGACTCGAAGAGC	GAGTTTTCGCTGGTCAAAGC	GCTTTCGCTTTTGTGCTACC
Sheep	GGAAAGCAGACTCGAAGAGC	GAGTTTTCGCTGGTCAAAGC	GCTTTCGCTTTTGTGCTACC
Oryx	GGAAAGCAGACTCGAAGAGC	GAGTTTTCGCTGGTCAAAGC	GCTTTCGCTTTTGTGCTA**T**C
Giraffe	GGAAAGCAGACTCGAAGAGC	GAGTTTTCGCTGGTCAAAGC	GCTTTCGCTTTTGTGCTACC
Deer	GGAAAGCAGACTCGAAGAGC	GAGTTTTCGCTGGTCAAAGC	GCTTTCGC**G**TTTGTGCTACC
Pig	GGA**GC**GCAGA**G**TCGAAGAGC	GAGTT**C**TCGCTGGTCAAAGC	GCTTTCGCTTTTGTGCTACC
Camel	GGA**GC**GCA**CC**CTCGAAGAGC	GAGTT**C**TCGCTGGTCAA**G**GC	GCTTTCGCTTTTGTGCTACC

Looking into sequence variation in cattle, nine SNPs were detected, eight in the coding region and one in the 3’UTR (Table 3), corresponding to 0.18 SNP per individual. Only four of these SNP produced amino acid changes (non-synonymous substitutions), three in the MHD region and the other in the C-terminal region. Of these amino acid changes in the MHD, three are predicted to be deleterious, and these were found in heterozygous individuals, carrying this mutation. Two of these SNPs changed from basic to polar, non-charged amino acids and one from polar, non-charged to the same type but a significant change in the structure. We found six additional individuals showing one and four individuals showing three heterozygous sites, all of these individuals belonging to the indicine and African breeds. When analyzing all the SNPs found here and those in the cattle database (Fig. 2), we found that the N-terminal, MHD and C-terminal showed similar percentages of nucleotides with non-synonymous substitutions (12.3, 12.8 and 12.5%, respectively), and were much higher than the percentage of synonymous substitutions (2.5, 2.6 and 4.9%, respectively).

**Table 3:**
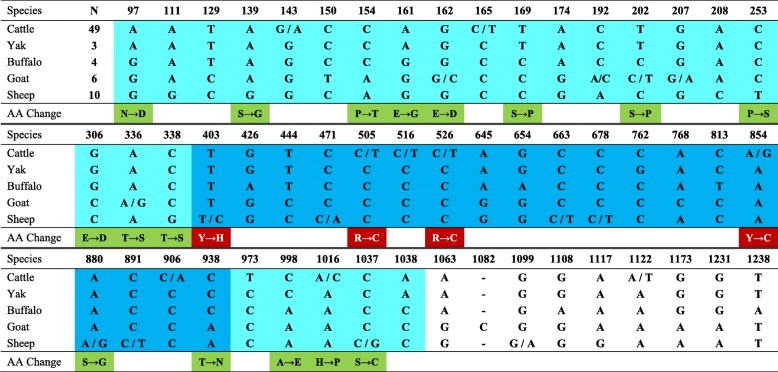
The nucleotide changes phylogeneticly important in the NDN sequences of cattle, yak, buffalo, goat and sheep, and the amino acid changes when appropriate

The numbering of the sequences was started at the transcription start site. The nucleotide in light blue represent the coding region while the darker blue represents the MAGE homology domain of the protein. N is the number of animals studied. The AA change shown in green are predicted to be neutral to the protein function, while those shown in red are predicted to be deleterious by PROVEAN software

In sheep, eight SNPs were also detected, which corresponds to 0.80 SNP per individual. Seven of the SNPs were located in the coding region and one in the 3’UTR, with three producing amino acid changes, two of which were also located in the MHD. One of these changes in the MHD was predicted to be deleterious, since it changed from a polar, non-charged to a basic amino acid, This mutation was also found in a heterozygous individual. No other heterozygous site was found in sheep. In goats, five SNPs were found, corresponding to 0.83 SNP per individual, and were localized in the coding region, but none at the MHD, and three of which produced amino acid changes. One heterozygous site was found among the individuals analyzed.

Of the 26 nucleotide changes that differentiate the species of Bovidae studied, only 11 produced amino acid changes (non-synonymous substitutions). The analysis of all the polymorphic sites found among these species shows that N-terminal region contain 16.2% of the nucleotides that were polymorphic, while 3.7% were polymorphic in the MHD region, and 3.5% in the C-terminal region. The analysis of the biochemical characteristics in the most common form of the proteins in each species show very little difference in molecular weight, pI, net charge, instability index, aliphatic index and GRAVY (Table 4) in the Bovidae species, except for sheep, which had a higher molecular weight, instability index and GRAVY. The proteins in pig, horse and dog did show differences in several characteristics, especially in molecular weight, net charge and GRAVY.

**Table 4:** Characteristics of the NDN proteins in Bovidae deducted from their sequences, using the web tool ProtParam

Species	Molecular Weight	pI	Net Charge	Instability Index	Aliphatic Index	GRAVY^a^
*Bos taurus*	36,590	8.88	+ 4	59.4	80.8	−0.414
Yak	36,590	8.88	+ 4	59.4	80.8	−0.414
Bison	36,590	8.88	+ 4	59.4	80.8	−0.414
Buffalo	36,597	8.67	+ 3	60.0	80.5	−0.425
Goat	36,597	8.67	+ 3	60.0	80.5	−0.425
Sheep	36,615	8.88	+ 4	57.40	80.5	−0.439
Pig	36,382	9.05	+5	59.6	81.1	−0.381
Horse	36,406	8.34	+ 2	61.2	79.0	−0.415
Dog	36,469	9.03	+5	62.5	77.8	−0.459

^a^grand average of hydropathicity

**Fig. 3:**
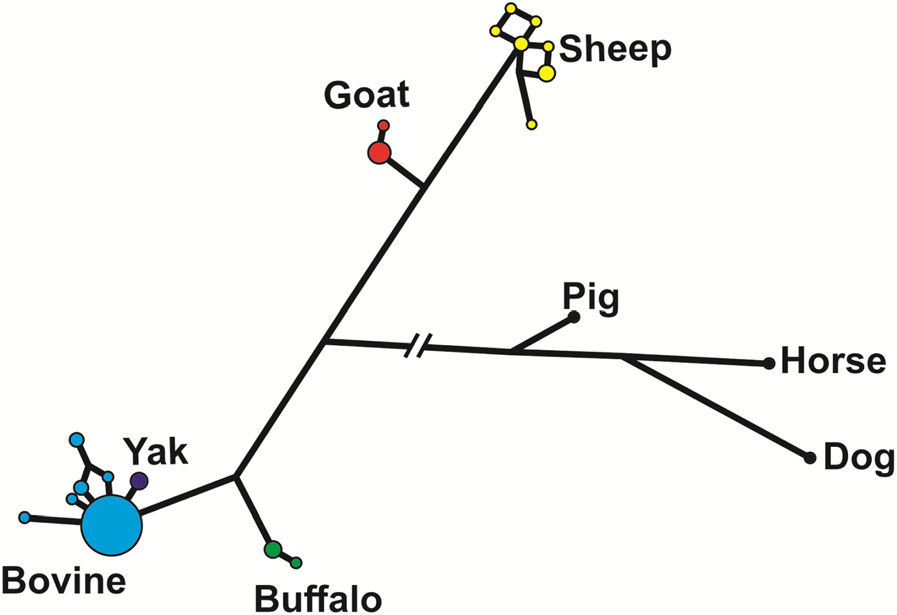
A median joining network of the genomic sequences using Network software (www.fluxus-engineering.com) of the NDN genes in species of Bovidae compared to pig, horse and dog. Circles represent haplotypes and have a size proportional to their frequencies

The median-joining network analysis of the genomic sequences of the species of Bovidae, including pig, horse and dog as outgroups (Fig. 3), shows that most of the haplotypes in cattle were identical, with only few very closely related haplotypes at a low frequency. The only yak haplotype found was also very closely related to the main cattle haplotype. On the contrary, sheep haplotypes were more diverse in spite of having only 10 individuals studied. In buffalos and goats, we found two haplotypes each, with a major and a minor one.

The phylogenetic analysis of the NDN sequences of the species of Bovidae and other species in the Cetartiodactyla was carried out which included the consensus of the 1194 bp sequence obtained here for cattle, yak, buffalo, sheep and goat, plus the upstream and downstream sequences of these species from the GenBank, to compare the entire sequence of the gene. In addition, we newly assembled and annotated the necdin gene by BLAST searching in the Sequence Read Archive (SRA) database, which contains next-generation sequencing data organized by the submitted sequencing project, in the following species: giraffe (SRA: SRX1624609, SRX1624612 and SRX1624614, Taxon ID: 439328), Oryx (SRA: SRX2880697, Taxon ID: 39411), white-tailed deer (SRA: SRX2056446, Taxon ID: 9874). The consensus sequences for the entire gene in these species were obtained by the alignment of the short reads with overlaps allowing each nucleotide position to be repeated at least 5 times. No polymorphism was found in any of the sequences.

**Fig. 4:**
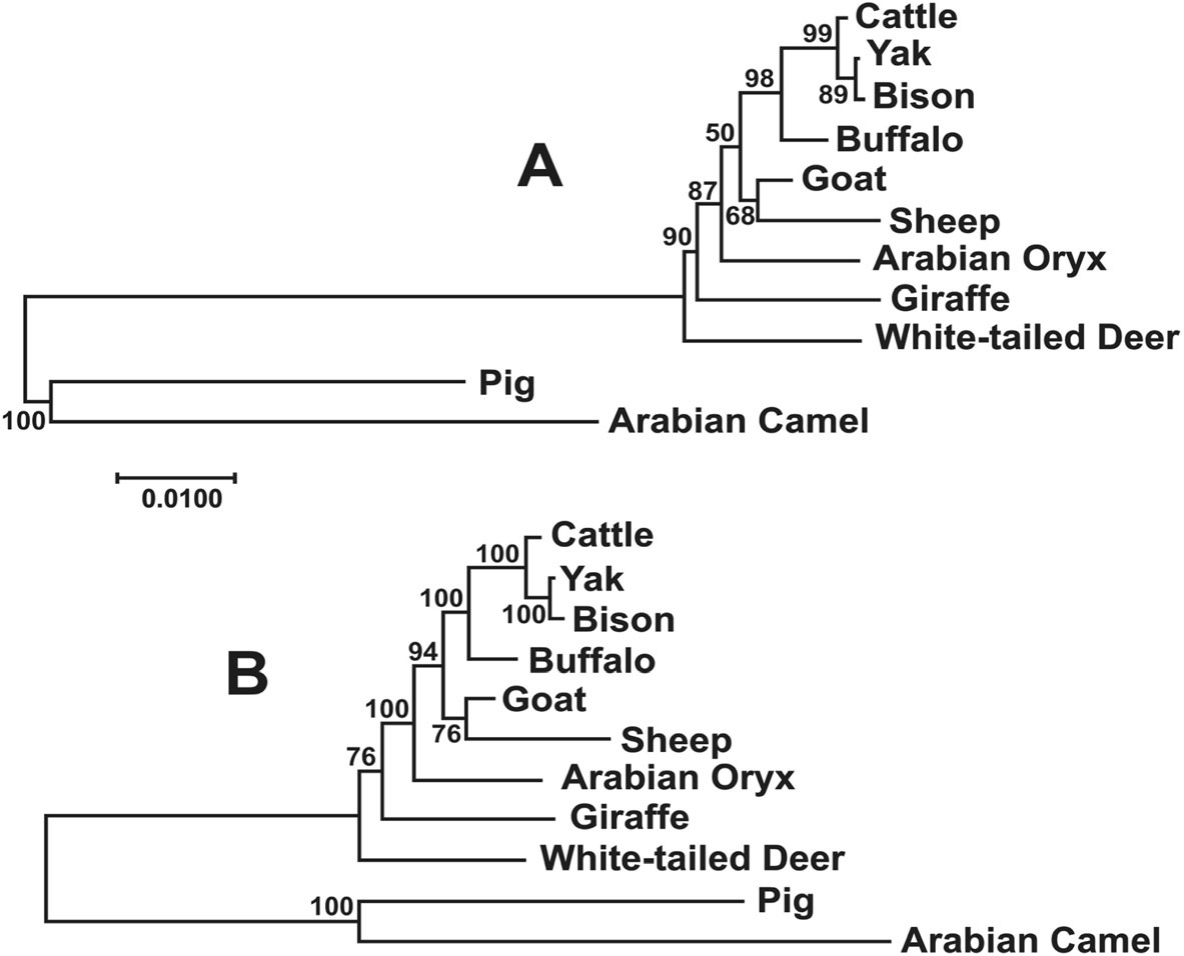
Phylogenetic analysis by Maximum Likelihood (**a**) and Bayesian (**b**) methods with the sequences obtained in this study and from GenBank on other Cetartiodactyla species. The percentages of replicate trees in which the associated taxa clustered together in the bootstrap test (1000 replicates for the ML tree and 200,000 iterations for the Bayesian tree) are shown next to the branches. The tree is drawn to scale with branch lengths in the same units as those of the evolutionary distances used to infer the phylogenetic tree which are the number of base substitutions per site. For the ML method, the tree with the highest log likelihood (− 5444.4659) is shown

The Maximum Likelihood and Bayesian phylogenetic analysis among the species of Bovidae produced trees almost identical (Fig. 4), although the statistical support for the Bayesian tree was higher. In this analysis, most branches showed the highest level of support (≥94%), with the exception of deer and sheep-goat, demonstrating the utility of these gene as a good phylogenetic marker in this group.

## Discussion

The use of primers localized in the most conserved regions of the 5′ and 3’ UTRs, should amplify all the species in the family Bovidae, since no variation or only one base change in the sequence of the primers was found in the species analyzed for this family, but should also amplify in the species of Giraffidae, Cervidae, Moschidae and Antilocaprinae. In addition, the use of the primer NDN-FCd and NDN-RCd, modified as shown, should amplify most the species from the other groups of Cetartiodactyla. This would be very useful to study the evolution of the sequence of the gene in all members of Cetartiodactyla, since it has been very difficult to stablish a detailed relationship among the different clades and to trace the pattern of evolution in this group, being one of the most diversified mammalian orders.

We found higher proportion of SNPs in sheep and goats than in cattle, even though we analyzed many more animals which were representatives of several cattle breeds originated in three different continents. The lower degree of sequence variation found for cattle, could be related to a bottleneck effect that has been suggested to have occurred during the domestication process, while no trace of bottleneck have been found during the domestication in sheep and goats [34]. However, we found several cattle individuals showing heterozygous sites, which is a sign of hybridization between breeds.

The amino acid sequence of the core functional domain of the NDN protein (aa 83–292) have been reported to be highly conserved between human and mouse (91% identity) suggesting evolutionary conservation due to a key biological function, but the sequence of the N-terminal region (aa 1–82) is less conserved, with about 60% identity [12]. This agrees with the identity of 87.1% in the N-terminal region, when comparing cattle and pig sequences, while identities of 94.1 and 93.7% are shown in the MHD and C-terminal regions. This also agrees with the fact that most of the nucleotide changes differentiating the species analyzed were found towards the N-terminal region. The fact that all of the predicted deleterious SNPs were found in the MHD region highlights the selection pressure that this sequence is under and the importance of this domain for the function of the NDN gene as well as other members of the type II MAGE genes, as this region has been shown to be highly conserved [35].

All the SNPs found here in the cattle breeds were novel for this species, most likely due to the higher representation of Asian and African breeds whose SNPs have not been well characterized. Interestingly, the SNPs at nucleotides 162 and 202 found in goats, producing non-synonymous changes, coincide with those reported for cattle (rs447229097 and rs434850213, respectively, www.ncbi.nlm.nih.gov/SNP). Another SNP found at nucleotide 891 in sheep, producing a synonymous change, was also reported in cattle (rs454519300). Thus, these represent SNPs conserved across these species, where balancing selection or a similar force could have maintained them because of a role in their evolution [36].

When analyzing the SNP density in the proximal region of bovine chromosome 21, Frischknecht et al. [21] found that there is a decreased SNP density at the beginning of BTA21 (less than 1000 SNPs per Mb) relative to other locations. The lack of detailed knowledge of the genomic organization, the imprinting status and transcriptional content precluded the analysis of candidate genes, in the study by Frischknecht et al. [21] and other genome-wide association studies. However, to this region, several QTLs have been associated to reproduction [20–22, 24] and early growth [21–23], among others. Thus, detailed studies on the genetic variation on the genes in this region, including NDN, are essential to pinpoint the location and functional analysis of the molecular mechanisms affecting these QTLs.

Studies of genomic imprinting in domestic livestock has focused on imprinted genes influencing fetal growth and development, which are associated with economically important production traits in cattle, sheep and pigs, since this can have major implications for the future of animal breeding, health and management [37], thus this study is a contribution towards the association of the variation found in NDN and the possible functional implication that it can produce.

## Conclusion

We found sufficient variation in the sequence of this gene among the individuals in all the studied species, besides the selection pressure this gene should be subjected to, due to the important functions in the organisms. Because NDN function is associated with cell growth, obesity and behavior, it can have effects in economically important traits in the production of cattle, yak, sheep, goat and buffalo. In addition, the phylogenetic trees constructed with the sequences of NDN showed the same pattern in Bovidae as previous report using nuclear genes, NDN has proven to be suitable for defining the evolutionary pattern in this group, which can be very useful in the phylogenetic reconstruction or to assess the genetic differentiation of the main groups or subgroups within the family, which have been difficult to determine and be useful for conservation genetics.
